# 

**Published:** 1921-03-05

**Authors:** 


### Forthcoming Events.

Royal Waterloo Hospital for Children and Women.—Annual Court, Wednesday, March 16, 3 p.m. Speakers, the Rt. Hon. the Lord Mayor, Mr. J. Topham Richardson, and Sir Edward Cooper. The Lady Mayoress and the Sheriffs of London, with their ladies, are expected to be present.

Friday, March 18, 4.30 p.m.—Conference convened by Medical Officers of Schools' Association at the Medical Society's Rooms, 11 Chandos Street, W. 1, on "Punishment in the Schools."

### Matrons' and Sisters' Appointments.

West Herts Hospital, Hemel Hempstead.—Miss Mary Alexander Lumsden has been appointed sister. She was trained at St. George's Hospital, S.W., and at the Royal Crichton Institution, Dumfries. She has done private nursing in Edinburgh.

Holmesdale Cottage Hospital, Sevenoaks.—Miss Kathleen Wafer has been appointed sister. She was trained at the West Middlesex Hospital and the Infants' Hospital, Vincent Square, has been staff nurse, Q.A.I.M.N.S., and at the Cottage Hospital, High Wycombe, Bucks.

